# DEMDATA: The Austrian-Czech institutional long term care project – design and protocol of a two-centre cross sectional study

**DOI:** 10.1186/s12913-017-2244-x

**Published:** 2017-04-20

**Authors:** Stefanie Auer, Elisabeth Linsmayer, Anna Beránková, Patrick Pascher, Bernadette Firlinger, Doris Prischl, Paulina Ratajczak, Edith Span, Iva Holmerova

**Affiliations:** 1 Department for Clinical Neuroscience and Preventive Medicine, Danube University Krems, Dr.-Karl-Dorrek-Straße 30, 3500 Krems, Austria; 2 Faculty of Humanities, Charles University, Prague, Czech Republic; 3 MAS Alzheimerhilfe, Bad Ischl, Austria

**Keywords:** Nursing homes, Health parameters, dementia, Database

## Abstract

**Background:**

The organization of long-term care is one of the main challenges of public health and health policies in Europe and worldwide, especially in terms of care concepts for people with dementia. In Austria and the Czech Republic the majority of elderly institutionalized persons with dementia are cared for in nursing homes. It is however unclear, how many persons living in nursing homes in Austria and in the Czech Republic are suffering from cognitive impairment and dementia. In addition, basic information on the nutritional status, the status of mobility and the medication prescription patterns are often missing. To facilitate new effective and evidenced based care concepts, basic epidemiological data are in urgent need. Thus, DEMDATA was initiated to provide important basic data on persons living in nursing homes in Austria and the Czech Republic for future care planning.

**Methods:**

DEMDATA is a multicentre mixed methods cross-sectional study. Stratified and randomly drawn nursing homes in Austria and the Czech Republic are surveyed. The study protocol used in both study centres assesses four different domains: a) Resident, b) Care team, c) Relative and d) Environmental Factors. Resident’s data include among others health status, cognition, dementia, mobility, nutrition, behavioural symptoms, pain intensity and quality of life. A minimum of 500 residents per country are included into the study (*N* = 1000 residents). The care team is asked about the use of the person-centred care and their burden. The relatives are asked about the number of visits and proxy-rate the quality of life of their family member. All staff employed in the nursing homes, all residents and relatives can voluntary take part in the study. The environmental factors include among others the organisational category of the nursing home, number of residents, number of rooms, social activities and the care concept. The project started in March 2016 and will be concluded in February 2018.

**Discussion:**

DEMDATA will provide important epidemiological data on four different nursing home domains in Austria and the Czech Republic, with a focus on the prevalence of dementia in this population. Thereby supplying decision and policy makers with important foundation for future care planning.

## Background

There are currently 46,8 million persons living with dementia worldwide [1]. Alzheimer Europe estimates that there are currently 145.431 persons with dementia living in Austria and 143.309 in the Czech Republic [2]. A rapid and continuous growth in an aging population [3] suggests that these numbers will double every 20 years [1]. This also entails an increase in persons living in nursing homes. Even though, there is a political plan to increase community care over institutional care, [4] the need for institutional Long term Care (iLTC) will remain over the next years. The organization of long-term care (LTC) including iLTC is one of the main challenges of public health and health policies in Europe [5–7] and worldwide [1], especially in terms of care concepts for people with dementia [8, 9]. The majority of elderly institutionalized persons from Austria and the Czech Republic with dementia living in iLTC are cared for in nursing homes. It is unclear however, how many persons living in nursing homes in Austria and in the Czech Republic are suffering from cognitive impairment and dementia. Studies conducted in other European countries produced diverse prevalence results. For example, in a large Swedish Study with 4831 studied nursing home residents, the prevalence of cognitive impairment was 67% [10]. In another study performed in nursing homes in Ireland, 58% percent had a dementia diagnosis. 31.8% had no medical dementia diagnosis but scored in clinical tests within the range of possible dementia, resulting in a total prevalence of 89.8% [8]. Considering the low diagnosis rate in some Austrian regions for home dwelling persons with dementia, the percentage of undiagnosed persons living in Austrian nursing homes may even be higher in some Austrian institutions [11]. Since an appropriate diagnosis is necessary for attending to the special medical and health care needs of this population, [9, 12] there is an urgent need for basic epidemiological data. The substantial increase of persons with dementia in nursing homes and the increased awareness of the needs of persons with dementia raises several challenges and requires either adaptations to current care practices or the development of alternative care concepts. Various approaches, most of them based on the concept of person-centred care [13] have been considered, but no convincing advantages of one care model over another could be found so far [14–16]. The results of different studies suggest a multifactorial approach to the development of specialized care models for persons with dementia, taking needs of residents, care teams and family members as well as environmental and psychosocial factors into account [17, 18]. The complex care needs associated with dementia in the different disease stages [10] lead to a higher care dependency, resulting in an increased stress and burden level in care teams [4, 19–21]. Testad et al. [22] found a positive correlation between behavioural symptoms and staff burden. A study in the Netherlands demonstrated how behavioural symptoms correlate with multiple environmental and social factors such as number of persons living in a unit, staff/patient ratio, the presence of a walking area and possibilities of social contact [23]. There is growing evidence that, even among those working in specialist dementia services, the proportion of staff receiving dementia care training is low [24–26]. Although dementia is an important topic in elderly long- term care, there are other important issues, such as infections [27] falls [28], pain management [29, 30] and the health status in general. There is also little knowledge on the potential for the improvement of medical services and the application of non-pharmacological therapies. The therapeutic nihilism in these environments is known to a certain extent but the potential has rarely been explored [31]. The lack of reliable information on these issues, as well as factors influencing the burden and stress of the care team is hindering the development of new innovative and cost effective care concepts [4].

DEMDATA was initiated to provide important basic data on persons with dementia living in nursing homes in Austria and the Czech Republic for future care planning. The goal of DEMDATA is to collect epidemiological data on four domains: a) Resident, b) Care team, c) Relative and d) Environmental Factors. This paper describes the main components of the DEMDATA study design, the study protocol for the quantitative data collection and the data management plan. The protocol for the qualitative data collection process will be described in a separate publication.

## Methods/Design

### Study design

DEMDATA is a multicentre mixed methods cross-sectional study. Stratified and randomly drawn nursing homes in Austria and the Czech Republic are surveyed. The project started in March 2016 and will end in February 2018. A minimum of 500 residents per country are included into the study (Total *N* = 1000 residents). This number has been estimated as a representative sample, as the population of nursing homes is relatively homogenous compared to community dwelling populations. The project is divided into four main study phases: Phase 1: Development and piloting of a common study protocol, Phase 2: Quantitative Data collection, Phase 3: Qualitative data collection, Phase 4: Analysis of data and publication of results.

A common working definition for the term ‘nursing home’ was agreed upon, as cross-country differences in institutionalized elderly care and different ideas on what necessary features constitute a nursing home exist. According to Sanford et al. [32], nursing homes are characterized by the following five parameters: (1) provides 24-h functional support for people who require assistance with activities of daily living (ADL)/instrumental activities of daily living (IADL) and have identified health needs; (2) may or may not be staffed with health care professionals; (3) provides long term care and/or rehabilitation as part of hospital avoidance or facilitate early hospital discharge; (4) does not function as a hospital ward and is not hospital based; (5) may play a role in providing palliative and/or hospice care at end of life.

There are currently 886 nursing homes in Austria and 822 in the Czech Republic. In Austria, there are three categories of nursing homes: (1) Homes under the administration of the federal state. In Upper Austria, these homes are managed by a commissioned organisation called *Sozialhilfeverband* (Social Help Organisation), (2) Homes under the administration of the municipality and (3) private homes of religious organizations or for profit organizations. To be admitted to a nursing home in Austria, a person needs to have a minimum level of care. The Austrian care system has seven levels of care. Each care level is defined by hours of care per month and person. Potential nursing home residents need to be ranked at a minimum level of 3, which is defined as: a person needing at least a care effort of 120 h/month.

There are 2 categories of nursing homes in the Czech Republic: a) Homes for seniors and b) Homes with a special regime. Each of these can be further organised in 3 categories: 1) Homes under the administration of the municipality, 2) private homes under the administration of for profit organizations, and 3) homes under the administration of non-governmental or religious organisations (Non-profit).

In order to minimize travel expenses for the research staff, three geographical areas for data collection were selected. In Austria, the federal state of Upper Austria with an area of 11.979,91 km^2^ and 1.453.733 inhabitants was selected. For the Czech Republic, the federal state Central Bohemia and Prague were selected. Central Bohemia has an area of 11.014,97 km^2^ and 1.291.816 inhabitants, Prague has an area of 496 km^2^ and 1.267.449 inhabitants.

According to an official list of the federal state of Upper Austria, 137 nursing homes are currently registered in Upper Austria. 159 nursing homes are registered in Central Bohemia and the Prague region.

Inclusion criteria for the residents are permanently living in the selected nursing home (independently of the length of stay) and a signed informed consent. All residents who have an acute health crisis (i.e. intensive care) or are in the process of dying will be excluded from the study. All care team members, administrative employees of the nursing homes, as well as relatives of the residents take part in the study on a voluntarily basis.

### Sampling procedure

#### Austria

**Fig. 1:**
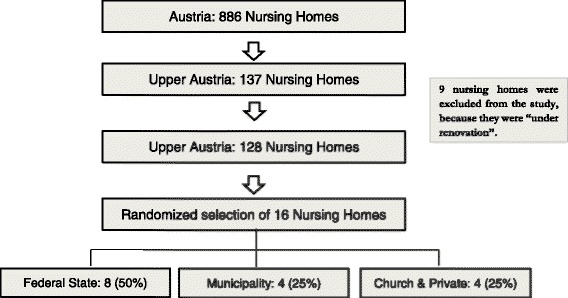
Study population & selection method in Austria

The 137 nursing homes of Upper Austria (from a total of 886 nursing home in Austria) formed the basis for the Austrian randomized selection process (see Fig. 1)﻿. Nine homes had to be excluded from the study, because they were “under renovation”. The remaining Austrian sample of 128 nursing homes includes facilities with 60–120 residents. From the remaining 128 homes, 82 nursing homes are run by the federal state, 21 are under municipal administration and 25 are organized by the church or private organizations. Each nursing home was assigned an identification number (ID). All numbers were written on separate pieces of paper and folded, concealing the ID number. The folded papers where then placed in different bowls representing the three organisational categories. Taking the drop outs into consideration, 16 nursing homes were randomly selected in a stratified fashion from the bowls (50% federal state; 25% municipality, 25% church/private). With this method, 16 nursing homes (8 federal state homes, and 4 municipal homes and 4 privately run homes) were randomly drawn. Of these 16 homes, seven nursing homes (five nursing homes run by the federal state, one home run by municipality and one home run by the church) consented to participate. In total, 644 residents are included in the Austrian sample.

#### Czech Republic

**Fig. 2:**
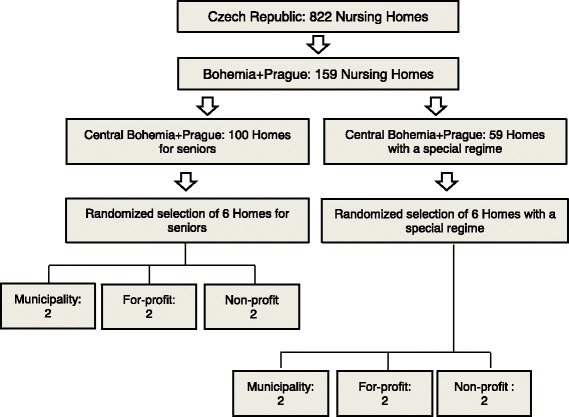
Study population & selection method in the Czech Republic

The 159 nursing homes (see Fig. 2) of Central Bohemia and the Prague region (from a total of 822 nursing homes in the Czech Republic) formed the basis for the Czech randomized selection process (100 homes of the category “homes for seniors” and 59 homes of the category “homes with a special regime”). Using a similar method as the Austrian team, 12 randomly selected homes were drawn (6 homes for seniors and 6 homes with a special regime) covering each sub-category (municipal, for-profit and non-profit). In the category “homes for seniors”, 6 nursing homes (3 municipal, 1 for-profit and 1 non-profit) consented to participate. In the category “homes with a special regime” four nursing homes (2 municipal, 1 for-profit and 1 non-profit) consented to participate. The resulting sample is very diverse in size – the smallest home provides services for 8 residents, whereas the biggest home provides care for 260 residents. In total, 570 residents are included in the Czech sample.

### Study protocol

The study protocol used in both study centres assesses four different domains: a) Resident, b) Care team, c) Relative and d) Environmental Factors. ﻿In Table 1, study instruments and data sources are listed.

**Table 1:** Instruments of the study protocol

Measurement/Variable	Measurement instrument/Items
Resident	
Dementia stage	Global Deterioration Scale (GDS)
Cognition	Brief Cognitive Rating Scale (BCRS)
	Mini Mental Status Examination (MMSE)
	Clock-drawing Test
Functioning	Functional Assessment Staging Test (FAST)
	KATZ Index of Independence in Activities of Daily Living
Mobility	Timed Up and Go Test
Nutrition	Mini Nutritional Assessment (MNA, short form)
Pain	Visual Analog Scale for Pain (VAS)
	Pain Assessment in Advanced dementia (PAINAD)
Behaviour	Behavioural Pathology in Alzheimer’s disease Frequency Weighted Scale (BEHAVE-AD-FS)
	Empirical Behavioural Pathology in Alzheimer’s disease Rating Scale (E-BEHAVE-AD)
Quality of Life	Quality of Life in Alzheimer’s disease Scale (QOL-AD participant version)
Sociodemographic data	Age, sex, date of institutionalization, nationality
Medication	Type and dosage
Dental status	Prosthesis (yes/no), other
Number of falls in the last 6 months	Chart Review
Number of hospital stays	Chart Review
Needs Assessment	Focus groups and qualitative interviews^a^
Care Team	
Person-Centred Care	Person-Centred Care Assessment Tool (P-Cat)
Burden	Professional Care Team Burden Scale (PCTB)
Sociodemographic data	Age, sex, occupational group
Needs Assessment	Focus groups and qualitative interviews^a^
Relative	
Sociodemographic data	Age, sex, relationship link to resident
Number of Visits	Daily, Every 2 or 3 days, once per week/month, other
Quality of Life	Quality of Life in Alzheimer’s disease Scale (QOL-AD family version)
Needs Assessment	Focus groups and qualitative interviews^a^
Environmental Factors	
Organisational Category	A: Federal State, Municipality, Church & private CZ: Municipality, For-profit, non-profit
Size of Institution	Number of organizational units, employees, rooms and residents
Room facilities	various listed facilities
Social Activities	Number per month, duration per unit, number of participants
Care Concept	Name or description of the care concept

^a^Qualitative part of the study will be described in a separate publication

#### Residents

For the assessment of dementia severity, the Global Deterioration Scale (GDS) [33] is used. Within the GDS, each stage is numbered from 1 to 7. The stages 1 to 3 are pre-dementia stages and stages 4 to 7 constitute dementia stages. To assess cognition, the Brief Cognitive Rating Scale [34], the Mini Mental State Examination (MMSE) [35] and the Clock drawing-test [36, 37] are used. The Brief Cognitive Rating Scale (BCRS) was constructed in accordance with the Global Deterioration scale and distinguishes seven stages, ranging from normal cognitive capacity (stage 1) to very severe cognitive deficits (stage 7). The capacities are measured on five subscales (concentration, short-term memory, long term memory, orientation and functioning/self-care) [34]. The MMSE, a well-known “gold standard”, assesses orientation, attention, immediate and short-term recall, language and the ability to follow simple verbal and written commands. The Clock drawing test provides a visual record of cognitive ability in an easy and time saving fashion.

The Functional Assessment Staging Test (FAST) [36] and the Katz-Index of Independence in Activities of Daily Living (Katz-Index) [37] are used to assess daily functioning. The FAST scale consists of a hierarchical list of typical symptoms, covering seven main functional levels (1 through 7) [38]. In addition, symptoms occurring in a non- ordinal fashion can be scored indicating excess disability in a multi-morbid nursing home population. The Katz-Index rates daily performance in six areas (bathing, dressing, toileting, transferring, continence and feeding), ranging from 1 to 6, six describing full functional capacity, 4 moderate impairment and ≥ 2 severe functional impairment [39].

The Timed Up and Go Test is used to assess mobility [40]. Persons are instructed to rise from an arm chair, walk three meters, turn, walk back and sit down again. The longer the duration, the lower the capacity of a person to move around.

The short form of the Mini Nutritional Assessment (MNA) [41] is used to assess the nutritional status of the resident. Scores greater than 12 indicate an acceptable nutritional status, scores from 8–12 point to a risk of malnutrition and scores lower than 8 indicate a state of malnutrition.

The Visual Analog Scale for Pain (VAS Pain) [42] is used to assess pain intensity. Residents are asked to rate their pain on a scale, ranging from 0 (no pain) to 10 (severe pain). Additionally, the Pain Assessment in Advanced Dementia (PAINAD) scale [43] is used for persons who are unable to reliably communicate their pain due to dementia or cognitive impairment. The participants are observed for two to five minutes during activities like bathing, turning or transferring. There are five categories (breathing, negative vocalization, facial expression, body language, consolability), which can be rated from 0 to 2. The sum of these scores results in the total score ranging from 0 (no pain) to 10 (severe pain).

Behavioural symptoms are assessed using the Behavioural Pathology in Alzheimer’s Disease Frequency Weighted Severity Scale (BEHAVE-AD-FW) [44] and the Empirical Behavioural Pathology in Alzheimer’s Disease Rating Scale (E-BEHAVE-AD) [45]. Both behavioural scales consist of 25 symptoms grouped into seven categories, which are rated from 0 to 3. The BEHAVE-AD-FW is assessed within an interview with a care team member, while the E-BEHAVE-AD is a direct observational scale scored within the assessment of the resident.

In order to receive an impression about the Quality of Life of the residents, the Quality of Life in Alzheimer’s disease (QOL-AD) [46] and the Euroquol (EQ) 5D-3 L scale [47] are used. The QOL-AD measures Quality of Life through 13 items on a 4-point Likert scale, ranging from 1 (poor) to 4 (excellent). To assess the perspective of the residents, the QOL-AD (participant-version) is used. The family members also rate the Quality of Life of the resident via the QOL-AD (family version). The EQ-5D-3 L consists of two parts. In the first part, the health status of the resident is assessed with five questions. In each question the resident can choose between three possible answers, ranging from Level 1 (no problem) to Level 3 (extreme problems). In the second part, the resident has to assess the health status on a visual analogue scale, ranging from 0 to 100 [48]. All used scales are valid, reliable and well established instruments.

Further information on the resident are derived from the care record: sex, age, date of institutionalization, citizenship, dementia diagnosis, other medical diagnoses, medication, dental status, number of falls and hospital-stays in the last 6 months.

#### Care team

Since Kitwood’s concept of person-centred-care has become a quality criterion for dementia care, the Person-Centred Care Assessment Tool (P-CAT) [49] has been included. The P-CAT questionnaire aims at evaluating the degree to which the care team experiences their work as being person-centred. It consists of 13 items, each rated on a 5-point Likert scale, ranging from ‘disagree completely’ to ‘agree completely’. A satisfactory construct validity and a good reliability have been reported for the P-CAT [49]. The Professional Care Team Burden (PCTB) [50] scale is used to assess the burden of the care team members. This ten item scale assesses three domains of burden: structural, organizational and subjective burden. The PCTB has an acceptable internal consistency (Cronbach’s Alpha, 0.779) and was well received by the staff [50]. Additionally, information on sex, age, occupational category and duration of the employment at the institution in question are collected.

#### Relatives

Data on the relative’s demographic characteristics (sex and age) and the relationship link to the resident are collected, as well as the frequency of visits. The quality of life of the resident is rated from the relative’s perspective with the QUOL-AD (family version).

#### Environmental factors

The following variables on environmental factors are collected for each nursing home: the category and size of the nursing home, the number of organizational units (care units), the number of employees (full- and part-time), organizational structure (management, experience of management), supervision (yes/no, mandatory) and the number and equipment of rooms per floor. Furthermore, information on the care concept of the nursing home and on the available social activities (number of activities per month, duration of a unit in minutes and the average number of participants for each activity) are collected.

### Study protocol pilot testing

The scales used in this study are used in their authorized translation in the respective language (German and Czech). If there was no translation available (for example the PCTB did not exist in the Czech language), a forward-backward translation was performed by native speakers.

Feasibility of the protocol with respect to lengths and order of the instruments was tested in a small pilot study with some residents, care team members and relatives of non-participating nursing homes. After the testing phase, small adjustments were made.

### Study organization

Before the start of the data collection a workshop for all participating nursing home managers is organized in both countries. In this workshop the project is introduced and organizational issues are discussed. Additionally, an information workshop in each nursing home is held, inviting all care team members and relatives as well as residents. In order to promote an optimal study flow a time schedule is agreed upon and the necessity for a project assistant for each nursing home, chosen from the respective nursing home care team, is discussed. The project assistants are crucial for a smooth assessment process within each nursing home. First, they help the researchers (i.e. clinical psychologists) in daily matters, such as recruitment of the care staff and relatives for the assessments, and second, support the data collection process (for example in the chart review). These assistants are hired for the duration of the presence of the research staff in each particular nursing home. An attendance time for the research team of 1 month was calculated for each nursing home.

### Data management plan

The collection process itself is strictly controlled by a Data Management Plan (DMP).

**Fig. 3:**
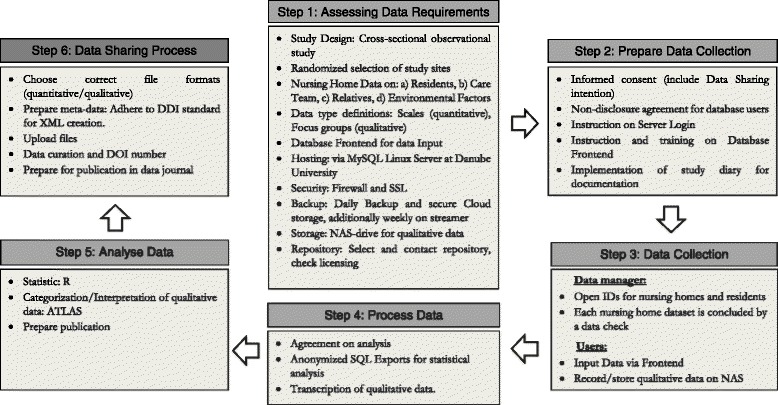
Data management plan

The DMP consists of six major steps (see Fig. 3). In step 1, all basic requirements for the data management process were clarified. A key component is the database at the Danube University, where all quantitative project data will be stored in an anonymized fashion. This not only facilitates an easier monitoring of the data regarding quality, but also enables various analysis methods. To minimize the risk of data loss a multilevel backup is in place. On a weekly basis the contents of the server are saved on a tape drive, a dump of the database is stored in a secure cloud and in addition, the database manager creates monthly dumps on an external drive, which is locked at a special compartment at the data manager’s office. As the collected data are of sensitive nature, various security measures have been put in place. The connections to the database and its interface are restricted and controlled by the data manager. In addition, the IT-department of the University monitors any incoming and outgoing connections.

Step 2 of the data management plan describes the user administration and security precautions, i.e. each database user has to sign a non-disclosure agreement. In order to guarantee data quality educational matters are described in Step 2 as well, thus in practice the data manager has to work closely with the field researchers. For example, they are instructed on how to correctly enter data into the frontend and on how to keep a research diary. This research diary is important to enhance the content of the meta-data. The actual data collection is coordinated in Step 3 of the data management plan. After the completion of the collection of quantitative data, the qualitative data collection is starting. These data are stored on the same server.

As step 4, a common quantitative data analysis is defined and the qualitative data are transcribed. In Step 5, the quantitative data are analysed, the qualitative data categorized and then the results will be summarized and synthesized for publications. Finally, the data will be made ready to share in Step 6 (funding for this process has been applied for).

### Statistical analysis plan and statistical considerations

The data will be exported from the database into CSV files via SQL-statements. Statistical analysis will then be performed via the open statistical software “R”. Descriptive statistics will be used to summarize the characteristics of the sample in the different countries and in the distinct nursing homes. A multilevel regression analysis will be performed per country to estimate the influence of different environmental and other factors (for example staffing) on the occurrence of neuropsychiatric symptoms. One particular issue will be the influence of the environments on the amount and severity of behavioural symptoms (e.g. the size of the nursing home, and patient/staff ratio). Another important issue will be the influence of the care team burden on the occurrence of behavioural problems. In addition, the correlation between the influence factors and the cognitive, functional und behaviour measurements will be investigated.

The distribution of the most important outcome variables and covariates will be used to check for outliers and differences between the two countries. Depending on the level of measurement Student’s *t*-test, Mann-Whitney *U* Test or *Chi* ^2^ Test will be used – especially for country comparison. Next, a pooled analysis will be performed and for the outcome variables, random effect models with the respective country as random factor will be conducted.

The statisticians of both project partners will cooperate on the definition of different topics of analysis.

## Discussion

DEMDATA will provide important epidemiological data on four different nursing home domains (resident, care team, relative and environmental factors) in Austria and the Czech Republic. A special focus is put on the prevalence of dementia in this nursing home population. If additional funding is granted, the data produced within this project will be prepared for data sharing and be made openly available in an anonymized fashion.

The strength of this multicentre mixed methods cross-sectional study is the size of the study population across countries, the harmonized study protocol with a broad range of variables and the storage of the data in a common database. By means of this data collection, key questions for future care planning can be addressed such as the prevalence of cognitive impairment and dementia within the nursing home milieu. Other important issues such as the influence of environmental factors on the development of behavioural symptoms – a factor which is extremely burdensome for resident, care team members and relatives alike. Potential country differences between Austria and the Czech Republic may demonstrate the necessity to modify old routines, structures and processes in the nursing homes in both countries and enable a cross-country dialogue. Currently, the scale, form and quality of long-term care provision in different countries is variable, suggesting considerable scope for sharing and learning from different national experiences [22]. Since there is still a significant paucity of knowledge in this area, the results of the qualitative part will serve for hypothesis building and recommendations for future research. Through understanding the unmet needs of residents with dementia, treatment plans and care concepts can be created which form the basis for nonpharmacological interventions [51]. There seems to be no doubt that specific care models for persons with dementia are required [20] and non-pharmacological interventions should be advanced. This study endeavours to enter into the discussions on these important matters providing further epidemiologic basic data. DEMDATA also has the potential to attract other countries to join this initiative for future projects, thus promoting further international co-operations. One of the major strengths of this study is, that the data will be assessed by trained researchers whereas other studies used untrained nursing staff for the assessment of residents and other variables [10]. Even though using untrained nursing home staff makes bigger data samples possible, working with trained researchers heightens the data quality. Since this project envisions to prepare the data for sharing, the quality of the produced data is of the utmost importance.

One of the main limitations of this study is the cross-sectional study design, which limits research into causal relationships between most of the factors assessed. However, with respect to the scarce data foundation currently available, the sole description and the comparison of several variables will enable the exploration of different correlations within and between different environments and countries. Thus, this data exploration will be an essential first step and further initiatives can start from a firm data foundation. The study protocol used in this study consist of different internationally well-established research instruments, some instruments however lack validation in the Czech and German language. In addition, considering the suspected high rate of cognitive impairment and dementia in this population, proxy instruments for the evaluation of resident variables are used. For that reason, the potential for self-reporting of needs will be explored in the qualitative research part of the study.

## Abbreviations

ADL: Activities of daily living
BCRS: Brief Cognitive Rating Scale
BEHAVE-AD-FW: Behavioural Pathology in Alzheimer’s disease Frequency Weighted
DMP: Data Management Plan
E-BEHAVE-AD: Empirical Behavioural Pathology in Alzheimer’s disease Rating Scale
FAST: Functional Assessment Staging Test
GDS: Global Deterioration Scale
IADL: Instrumental activities of daily living
ID: Identification number
iLTC: Institutional Long Term Care
LTC: Long Term Care
MMSE: Mini Mental Status Examination
MNA: Mini Nutritional Assessment
PAINAD: Pain Assessment in Advanced dementia
P-CAT: Person-Centred Care Assessment Tool
PCTB: Professional Care Team Burden Scale
QOL-AD: Quality of Life in Alzheimer’s disease Scale
VAS Pain: Visual Analog Scale for Pain
